# Critical Role of Endothelial Hydrogen Peroxide in Post-Ischemic Neovascularization

**DOI:** 10.1371/journal.pone.0057618

**Published:** 2013-03-05

**Authors:** Norifumi Urao, Varadarajan Sudhahar, Seok-Jo Kim, Gin-Fu Chen, Ronald D. McKinney, Georg Kojda, Tohru Fukai, Masuko Ushio-Fukai

**Affiliations:** 1 Department of Pharmacology, Center for Lung and Vascular Biology, Center for Cardiovascular Research, University of Illinois at Chicago, Chicago, Illinois, United States of America; 2 Departments of Medicine and Pharmacology, Center for Cardiovascular Research, University of Illinois at Chicago, Chicago, Illinois, United States of America; 3 Jesse Brown Veterans Affairs Medical Center, Chicago, Illinois, United States of America; 4 Institute of Pharmacology and Clinical Pharmacology, University Hospital Düsseldorf, Düsseldorf, Germany; University of Bristol, United Kingdom

## Abstract

**Background:**

Reactive oxygen species (ROS) play an important role in angiogenesis in endothelial cells (ECs) *in vitro* and neovascularization *in vivo*. However, little is known about the role of endogenous vascular hydrogen peroxide (H_2_O_2_) in postnatal neovascularization.

**Methodology/Principal Findings:**

We used Tie2-driven endothelial specific catalase transgenic mice (Cat-Tg mice) and hindlimb ischemia model to address the role of endogenous H_2_O_2_ in ECs in post-ischemic neovascularization *in vivo*. Here we show that Cat-Tg mice exhibit significant reduction in intracellular H_2_O_2_ in ECs, blood flow recovery, capillary formation, collateral remodeling with larger extent of tissue damage after hindlimb ischemia, as compared to wild-type (WT) littermates. In the early stage of ischemia-induced angiogenesis, Cat-Tg mice show a morphologically disorganized microvasculature. Vascular sprouting and tube elongation are significantly impaired in isolated aorta from Cat-Tg mice. Furthermore, Cat-Tg mice show a decrease in myeloid cell recruitment after hindlimb ischemia. Mechanistically, Cat-Tg mice show significant decrease in eNOS phosphorylation at Ser1177 as well as expression of redox-sensitive vascular cell adhesion molecule-1 (VCAM-1) and monocyte chemotactic protein-1 (MCP-1) in ischemic muscles, which is required for inflammatory cell recruitment to the ischemic tissues. We also observed impaired endothelium-dependent relaxation in resistant vessels from Cat-Tg mice.

**Conclusions/Significance:**

Endogenous ECs-derived H_2_O_2_ plays a critical role in reparative neovascularization in response to ischemia by upregulating adhesion molecules and activating eNOS in ECs. Redox-regulation in ECs is a potential therapeutic strategy for angiogenesis-dependent cardiovascular diseases.

## Introduction

Neovascularization in response to tissue ischemia or injury is an important adaptive mechanism that is involved in wound repair as well as ischemic heart and limb diseases. It depends on angiogenesis (a process of new vessel formation from pre-existing capillary-like endothelial cells (ECs)), arteriogenesis [1] and bone marrow (BM)-derived vascular progenitor cells [2]–[5]. Inflammatory cell infiltration into ischemic tissues, which is in part mediated by the adhesion molecules expressed in ECs, also plays an important role in ischemia-induced revascularization by releasing vascular endothelial growth factor (VEGF) [6]. VEGF induces angiogenesis by stimulating cell migration, proliferation and capillary tube formation in ECs. Thus, understanding the mechanisms by which vascular ECs regulate neovascularization *in vivo* is critically important for developing new therapeutic strategies for treatment of ischemic cardiovascular diseases.

Reactive oxygen species (ROS) such as superoxide anion (O_2_^•−^) and hydrogen peroxide (H_2_O_2_) play important roles in angiogenesis in cultured ECs as well as postnatal neovascularization *in vivo* [7]–[9]. There is doubled-edged effect of ROS whereby ROS at physiological levels mediate biological responses including angiogenesis [10], [11], whereas excess ROS levels in pathological conditions induce detrimental effects such as cell death, and impaired neovascularization [12]–[15]. The levels of ROS are determined by the balance of ROS generation and antioxidant enzyme activity. Although there are many sources of ROS, NADPH oxidase (Nox) is one of the major ROS generating enzymes involved in postnatal angiogenesis [7]–[9]. We previously demonstrated that Nox2-derived ROS play an important role in reparative neovascularization in response to hindlimb ischemia [11], [16]. Nox2 activation initially produces O_2_^•−^ that can be rapidly converted to H_2_O_2_ by superoxide dismutases (SODs) [17], [18], or reacts with nitric oxide (NO) to induce endothelial dysfunction [19]. Of note, H_2_O_2_ is chemically more stable than other ROS such as O_2_^•−^ and does not react with NO, thereby penetrating through the vascular wall. H_2_O_2_ is produced intracellularly in response to various stimuli, including cytokines and growth factors such as VEGF, and functions as a signaling molecule to mediate angiogenesis in ECs [20]. It can also accumulate extracellularly in the tissue and survive long enough to induce numerous paracrine functions, even in more distant cells. H_2_O_2_ is degraded to water by catalase [21], glutathione peroxidases (GPx) [22] and peroxiredoxin (Prx) systems [23]. Different from Nox2, Nox4 activation directly produces H_2_O_2_ into the cytosol due to its intracellular localization [7]. It is recently reported that endothelial-specific Nox4 overexpressing transgenic mice promote ischemia-induced angiogenesis [24], while global or conditional Nox4 knockout mice without tissue specificity inhibit this response [25]. Myeloid-specific catalase overexpression in mice show impaired post-ischemic neovascularization [26]. However, the specific role of endogenous H_2_O_2_ in ECs for postnatal neovascularization *in vivo* remains unknown.

In the present study, we used transgenic mice with endothelial-specific overexpression of human catalase [27], [28] to examine whether endogenous H_2_O_2_ in ECs is required for neovascularization following hindlimb ischemia. Here we demonstrate that endothelial H_2_O_2_ plays a critical role in angiogenesis, inflammatory cell recruitment and the early phase of vascular progenitor cell mobilization from the BM induced by ischemic injury. We also found that endogenously produced H_2_O_2_ contributes to not only new vessel formation but also endothelial function of resistant vessels.

## Methods

### Animals

Animal housing and study protocols were approved by the Animal Care and Institutional Biosafety Committee of University of Illinois at Chicago (ACC: 09–066, 09–067, 12–067 and 12–069), and the experiments were performed according to the Guide for the Care and Use of Laboratory Animals of the National Institutes of Health. Generation of Tie2-driven catalase transgenic mice has been reported [28]. Briefly, human catalase was inserted between murine Tie2-promotor and a 10-kb Tie2 intron fragment, designated as Tie2 enhancer. Founder mice showing approximately 100-fold higher catalase expression were crossed 10 times to C57BL/6 mice to generate pure C57BL/6 background [29]. The transgenic mice were bred and genotyped as described [28]. The transgenic mice (range 8–12 week-old) and sex-matched transgene negative littermate wild-type (WT) mice were used for each experiment.

### Mouse hindlimb ischemia model

Mice were subjected to unilateral hindlimb surgery as we described [11], [30], [31] and with a slight modification. To induce hindlimb ischemia, left femoral arteries were ligated at just distal of the branch point for profunda femoris and the proximal of the saphenous artery under anesthesia with ketamine (100 mg/kg) and xylazine (10 mg/kg). The artery between the ligations was removed and all of its branches were obliterated using an electrical coagulator. Buprenorphine at the rate of 0.1 mg/kg were given prior to the surgery and twice a day after the surgery for 3 days as analgesia. Mice with major bleeding or signs of infections were euthanized. Laser Doppler imaging for blood flow measurement was carried out with PeriScan PIM 3 System (Perimed) as described previously [31]. The recovery of blood flow was expressed as the ratio of foot perfusion with correction by the flow ratio measured before the surgery.

Ischemic and nonischemic muscles were harvested, fixed with 4% paraformaldehyde and frozen in Tissue Tek O.C.T. compound (Sakura Finetak). The sections of 7 μm thickness were stained with antibodies against mouse CD31 (BD Pharmingen) or F4/80 (Biolegend) followed by biotin-conjugated anti-rat IgG antibody (Vector Laboratory) and visualized by VECTOR NovaRED or VECTOR DAB following peroxidase labeling with VECTASTAIN Elite ABC Reagent (Vector Laboratories). Counterstaining with hematoxylin or eosin was performed. CD31 positive cell was counted in at least 3 different microscopic fields (x40) at the triangle region of gastrocnemius muscles [32]. Capillary density was expressed as the CD31 positive per muscle fiber. F4/80 positive cell infiltrated area in the region was defined as the fibers with at least three F4/80 positive cells around. Some tibialis anterior muscles and adductor muscles were stained with hematoxylin and eosin for morphological and morphometric analysis. Necrotic area was defined by fibro-adipose tissue infiltration and the existence of ghost muscle cells devoid of nuclei, as described previously in tibialis anterior muscles [31]. Collateral morphometry was performed in the center part of semimembranosus muscle of the adductor muscle series by tracing the inner and outer circumference of the vessels on the digital images by Image J software, as previously described [31], [33].

### Aortic ring assay

The thoracic aortas were freshly harvested and cleaned, then 1-mm segments were placed in 300 μL cold Matrigel (BD bioscience) in a 48-well plate. After 30 minutes in a 37°C incubator, EndoGro media/5% fetal bovine serum (Millipore) was added, and the medium was changed every other day. Capillary sprouting was counted by phase-contrast microscopy with the use of at least 12 segments of aorta from 3 mice per group. Aortic ring sprouts were analyzed carefully on the basis of morphological differences in growth between the endothelial sprouts and fibroblast sprouts based on greater thickness and a uniform pattern of growth. Sprout counting was confirmed with lectin staining (data not shown).

### Quantitative RT-PCR analysis

Total RNA was prepared from cells using TRI Reagent (Molecular Research Center) according to manufacturer's protocol. Reverse transcription was carried out using high capacity cDNA reverse transcription kit (Applied Biosystems). The real-time PCRs were run on an ABI Prism 7000 Sequence Detection System (Applied Biosystems) in the SYBR Green PCR kit and the QuantiTect Primer Assay (Qiagen) for specific genes.

### Peripheral blood analysis

Peripheral blood was collected and analyzed as described previously [31]. Total white blood cell was counted manually in the cell suspension from the buffy coat. Monocytes were defined as CD11b^+^/Ly-6G^−^ cells by multicolor FACS analysis. For vascular progenitor cells, Sca-1 and Flk-1 were used. All the antibodies were obtained from BD bioscience. A flow cytometer (DAKO ADP Cyan) equipped with Summit software (DAKO) and FlowJo 7.6 software (Tree Star) was used for population analysis.

### Redox status and H_2_O_2_ measurement

For intracellular redox status_,_ cell samples and cultured cells were stained with 10 μM for 6 minutes or 5 μM for 15 minutes incubation of 5-(and-6)-chloromethyl-2′,7′-dichlorodihydrofluorescein diacetate, acetyl ester (CM-H2DCFDA, Invitrogen) at 37°C, respectively and analyzed by FACS or laser confocal microscopy. Cell suspension from collagenase-digested tissue samples were used to detect H_2_O_2_ levels in ECs combined with surface marker staining [34] with anti-CD31 and anti-CD45 antibodies (both BD bioscience). Extracellular H_2_O_2_ produced from non-ischemic and ischemic tibialis anterior muscles was measured by Amplex Red assay with Amplex Ultra Red (Invitrogen), according to manufacturer's instruction. The values were standardized with tissue dry weights.

### Western blotting

Western blot analyses were performed as described [35] with modifications. Mice were perfused with cold phosphate buffer saline. Muscle samples were harvested and frozen in liquid nitrogen. Muscle samples were crushed and lysed with RIPA lysis buffer (5 mM Tris-HCl (pH 7.6), 150 mM NaCl, 1% NP-40, 1% sodium deoxycholate, 0.1% SDS) followed by brief sonication. Total protein was measured by BCA protein assay (Thermo scientific). Equal amount of protein was separated by SDS-PAGE. Following primary antibodies were used; p-eNOS (Ser1177) (9571; Cell Signaling), eNOS (610297; BD bioscience), p-Akt (Ser473)(4060; Cell Signaling), Akt (4691; Cell Signaling), ERK1/2 (9102; Cell Signaling) and VEGF (A-20; Santa Cruz).

### Vascular reactivity studies

Isometric tension of mesenteric resistance arteries were measured using wire myograph (Model 610 M, Danish Myo Technology, Denmark) as described [36]. Briefly, the first or second order branches of resistance arteries were isolated from mice mesenteric bed, cut into ∼2 mm segment and stored in cold Krebs Physiological Salt Solution (PSS) (119.0 mM NaCl, 25.0 mM NaHCO_3_, 4.6 mM KCl, 1.2 mM MgSO_4_, 1.8 mM CaCl_2_, 11.0 mM glucose) at pH 7.4. The vessel were mounted in between two hook using tungsten wire (25 µm in diameter) in organ chamber which containing Krebs PSS bubbled with gas mixture containing 5% CO_2_ and 95% O_2_ at 37°C. Basal tension was set on arteries stretched to L100, where L100 is defined as the circumference of the relaxed artery exposed to a transmural pressure of 100 mmHg and equilibrated for 1 hr. After equilibration, the arteries were exposed to high concentration of KCl (80 mM) and 10 μM noradrenalin for 2–3 min until reproducible maximal contractions occur. The α-adrenergic receptor agonist, phenylephrine was added to increase basal tension to 60% to 80% of maximal KCl contraction. Cumulative concentration (0.01–10 μM) of acetylcholine (ACh) were added to the bathing solution every 5 min. At the end of the each experiment, cumulative concentration of sodium nitroprusside (0.01–10 μM) was added to the bath to demonstrate the intact smooth muscle function. Results are expressed as percent relaxation of the phenylephrine-treated arteries, with 100% relaxation representing basal tension.

### Statistical analysis

All values were expressed as means. Blood flow recovery in the ischemic hindlimb was compared between the two groups by two-way repeated measures ANOVA, followed by Bonferroni post hoc analysis. Comparison between groups was analyzed by unpaired Student 2-tailed t test (2 groups) or ANOVA for experiments with more than 2 subgroups followed by Bonferroni post hoc analysis. P<0.05 was considered statistically significant.

## Results

### Neovascularization is impaired in Cat-Tg mice following hindlimb ischemia

To examine the role of endogenous H_2_O_2_ in ECs in post-ischemic neovascularization, we used previously established transgenic mice with endothelial-specific overexpression of human catalase driven by the Tie2 promoter [37] (Cat-Tg mice). We previously demonstrated the endothelial-specific catalase overexpression and reduction of EC-derived H_2_O_2_ in vascular cells or blood vessels in Cat-Tg mice [27], [28]. In this study, we confirmed the increase in human catalase mRNA in aorta (Figure S1A) and catalase protein expression in the cultured ECs in Cat-Tg mice (Figure S1B). Furthermore, VEGF-induced increase in intracellular oxidation state, as measured by DCF-DA fluorescence analysis [38], [39], was significantly inhibited in cultured ECs derived from Cat-Tg mice, as compared to WT-ECs (Figure S1C).

**Figure 1:**
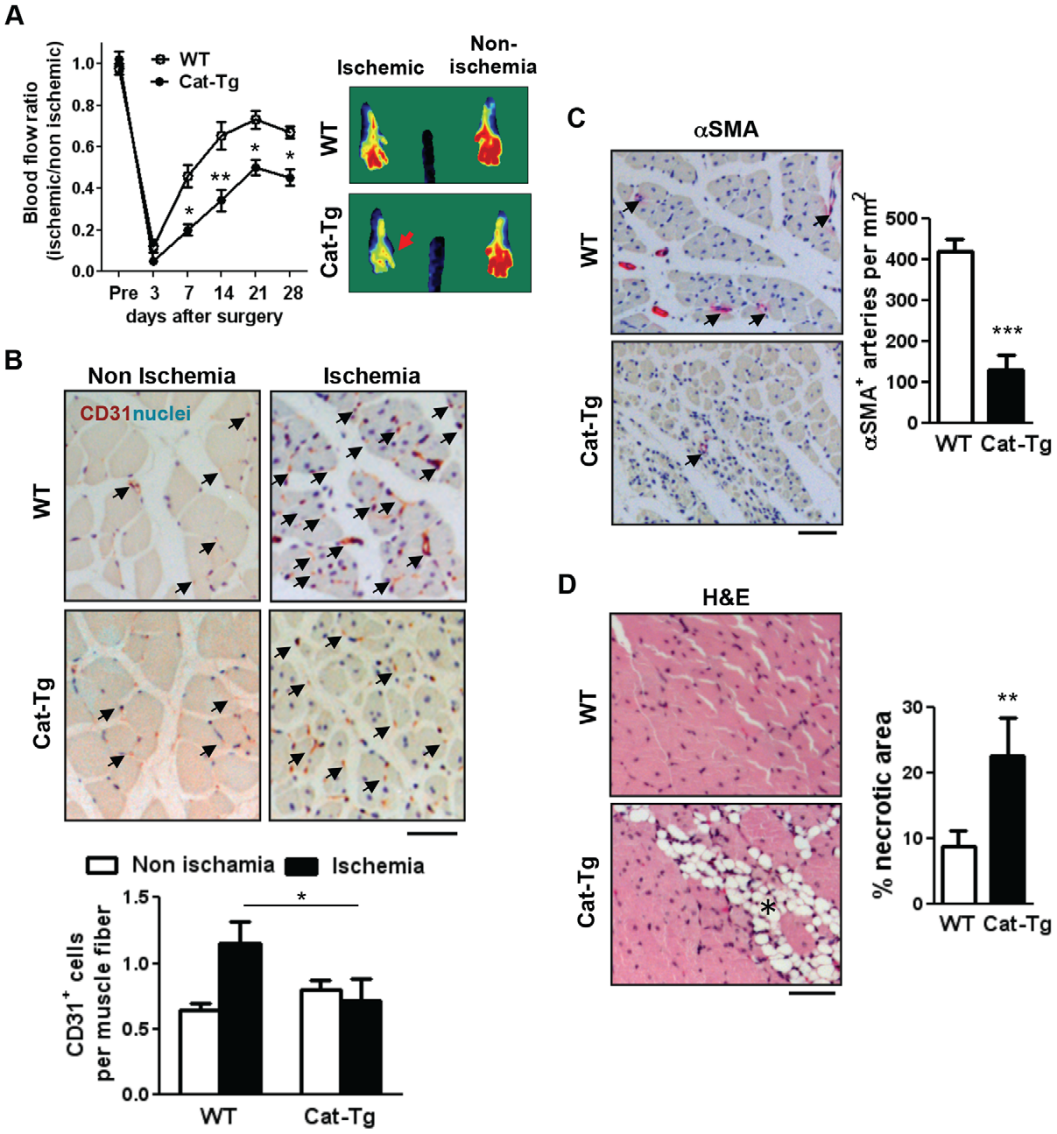
Endothelial catalase overexpression impairs post-ischemic neovascularization. **A**, Wild-type (WT) and Tie2-driven catalase transgenic (Cat-Tg) mice were subjected to hindlimb ischemia. Blood flow recovery was measured by relative values of foot perfusion between ischemic and non-ischemic legs (WT n = 9, Cat-Tg n = 7,). Representative laser Doppler images at day 28 (right panels). **B**, capillary formation in the ischemic gastrocnemius muscles were analyzed by immunostaining with an endothelial-marker CD31 (red-brown and arrows) and quantified as capillaries per muscle fibers in the graph (n = 4 mice per group and bar = 50 μm). **C**, the density of arterioles are measured by αSMA staining (red and arrows) on the ischemic gastrocnemius muscles (n = 4 mice per group and bar = 50 μm). **D**, tissue repair after ischemic injury was examined in the ischemic gastrocnemius muscles with haematoxylin and eosin (H&E) staining which show necrotic regions with fibro-adipose tissue infiltration (asterisk in the image) as a sign of impaired or delayed repair process (n = 4 mice per group and bar = 50 μm). All data shown are mean+SE (*p<0.05 and **p<0.01).

We used a hindlimb ischemia model to examine the postnatal neovascularization in WT and Cat-Tg mice. LDBF analysis in ischemic and non-ischemic legs showed that the blood flow recovery after femoral artery excision was significantly inhibited in Cat-Tg mice (Figure 1A). This was associated with a decrease in the numbers of CD31 positive capillaries (Figure 1B) and α-smooth muscle actin positive arterioles (Figure 1C) in the ischemic region of gastrocnemius muscles at 28 day after injury. Histological analysis revealed that necrotic area was significantly increased in ischemic tissues of Cat-Tg mice (Figure 1D).

**Figure 2:**
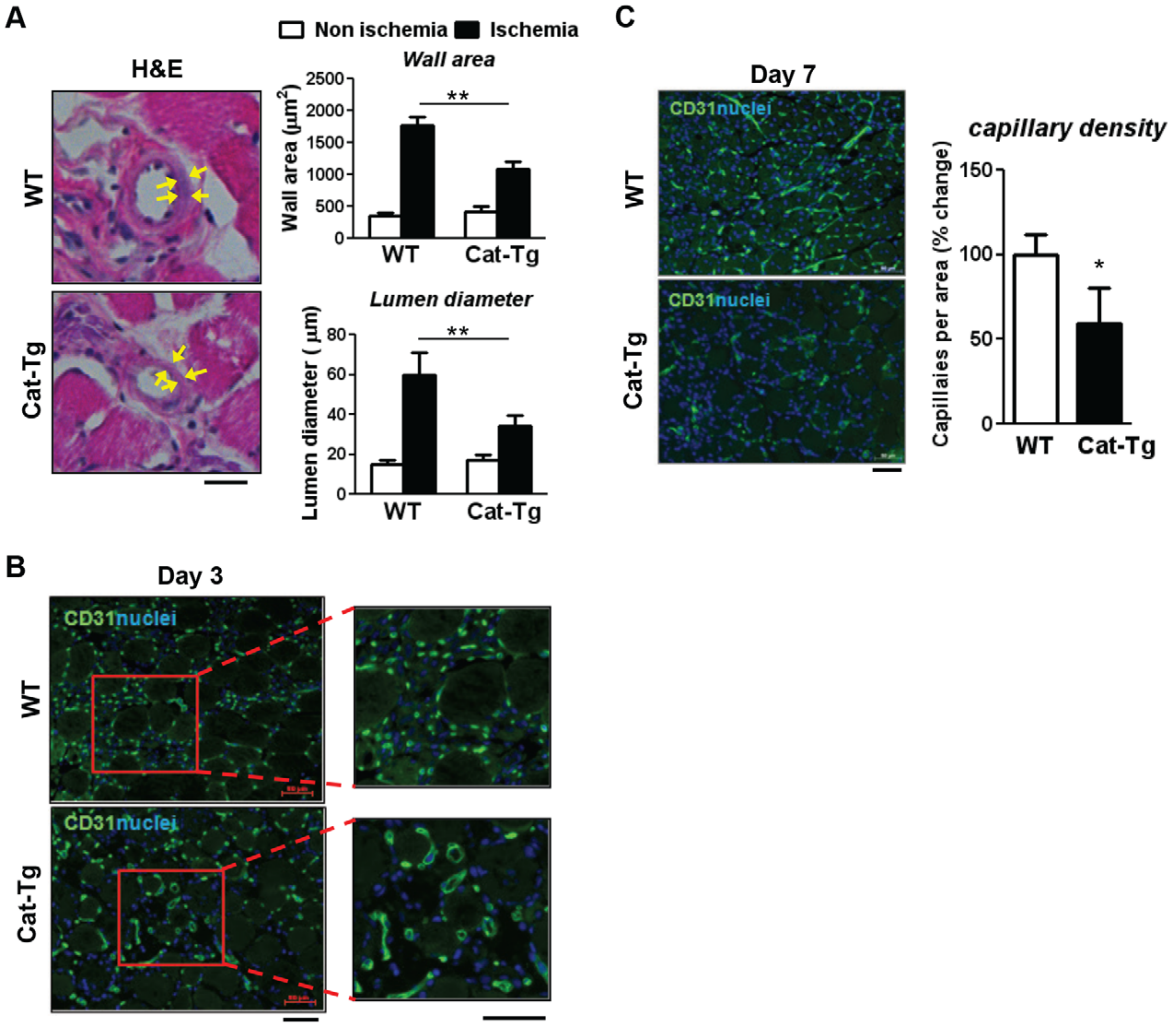
Endothelial catalase overexpression impairs collateral remodeling and stabilization of vessels undergoing neovascularization. **A**, collateral remodelling after hindlimb ischemia was analyzed in the same anatomical arteries localized at semimembranosus muscles in the upper limbs between wild-type (WT) and Tie2-driven catalase transgenic (Cat-Tg) mice. The luminal diameter and wall area are calculated from the measurements of luminal and perivascular tracing. Arrows indicate collateral wall. **B**, ischemic gastrocnemius muscles were analyzed for the morphology of vessels at day 3 with immunostaining for an endothelial-marker, CD31 (green). Nuclei were visualized by 4′,6-diamidino-2-phenylindole (DAPI) (blue). Magnified images show that morphologically disorganized vessels with varying size, enlarged lumen and irregular shape are often seen in Cat-Tg mice. C, capillary densities in the same region as B were analyzed by CD31 staining at day 7. Bars indicate 20 μm in A, and 50 μm in B and C. All data shown are mean+SE (n = 3–4 mice per group, *p<0.05 and **p<0.01).

We next analyzed the collateral remodeling in the upper limbs using H&E staining and found that collateral lumen diameter and wall area were increased in WT mice at day 7 after hindlimb ischemia, which was significantly inhibited in Cat-Tg mice (Figure 2A). There was no difference in the size of collateral arteries in non-ischemic contralateral limbs between WT and Cat-Tg mice. Additionally, immunofluorescence analysis with CD31 antibody in the lower limb at day 3 after ischemia showed that Cat-Tg mice had a morphologically disorganized newly formed microvasculature characterized by varying size, enlarged lumen and irregular shape (Figure 2B), which is often seen in tumor angiogenesis with impaired vessel normalization or maturation [40]. Subsequently, this was followed by a significant decrease in the capillary density at day 7 after ischemia in Cat-Tg mice (Figure 2C). Thus, endogenous endothelium-derived H_2_O_2_ is required for post-ischemic neovascularization.

### Endothelial catalase overexpression reduces vessel sprouting and tube elongation *ex vivo*

**Figure 3:**
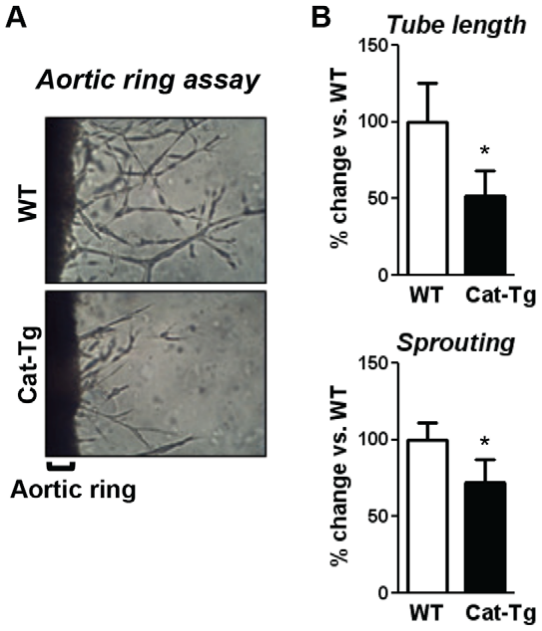
Endothelial catalase overexpression impairs vessel sprouting and tube elongation in *ex vivo* aortic ring assay. Aortas were harvested from Wild-type (WT) and Tie2-driven catalase transgenic (Cat-Tg) mice and cultured in Matrigel for 7 days. Capillary sprouts and average tube length were measured in 5 rings from each aorta under microscopy (n = 3 mice per group and *p<0.05). Data shown are mean+SE.

To determine further the role of endothelial H_2_O_2_ in capillary sprouting from pre-existing vessels, we performed the mouse aortic ring assay *ex vivo* using isolated aorta from Cat-Tg and WT mice. This assay is enabling us to examine the early angiogenic processes without contribution of systemic factors such as blood flow, blood pressure and homeostatic regulation [41]. As shown in Figure 3, isolated aorta from Cat-Tg mice cultured in Matrigel with VEGF exhibited impaired capillary sprouting and tube elongation, compared with control.

### Myeloid cell recruitment is inhibited in Cat-Tg mice following hindlimb ischemia

**Figure 4:**
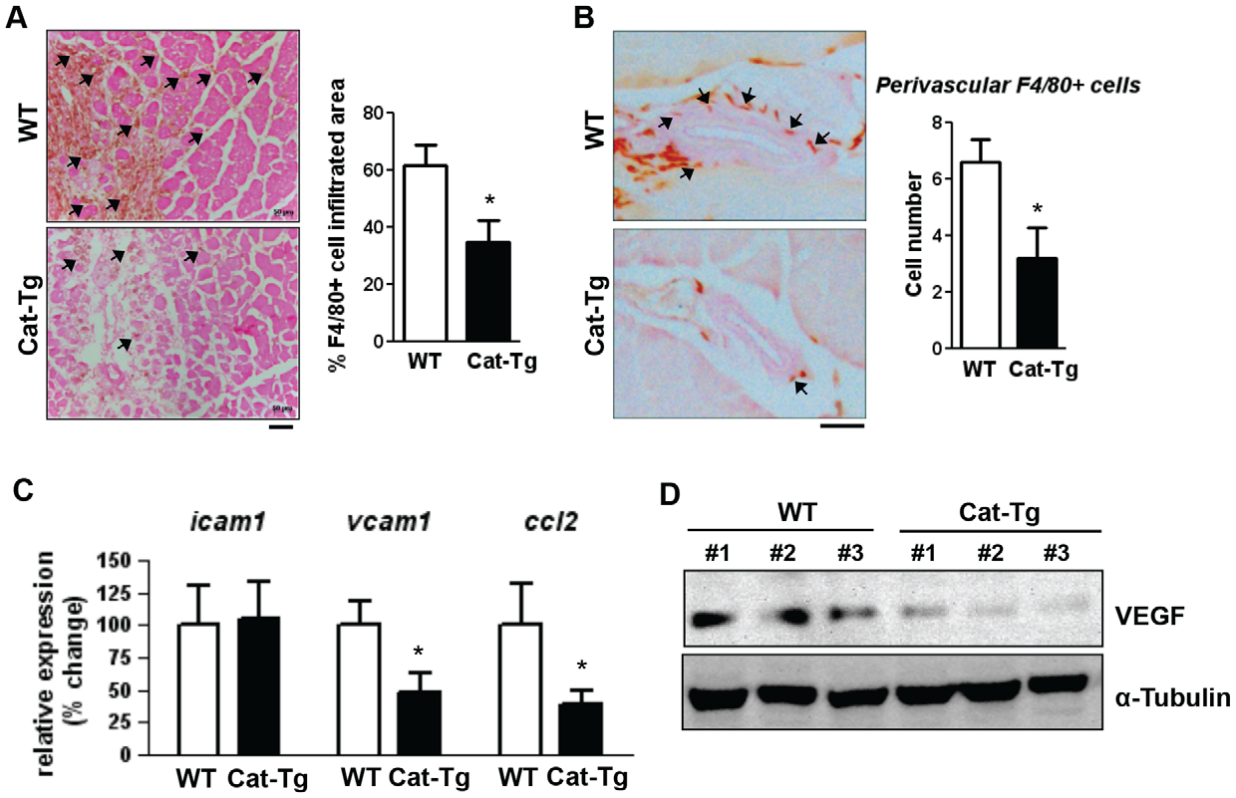
Endothelial catalase overexpression decreases the recruitment of F4/80+ myeloid cells to the ischemic tissue. **A**, the ischemic area of gastrocnemius muscles from wild-type (WT) and Tie2-driven catalase transgenic (Cat-Tg) mice at day 7 was analyzed for myeloid cell recruitment with immunostaining for F4/80 (brown and arrows). The percentage of F4/80+ cell infiltrated area in the damaged region of gastrocnemius muscles is shown (n = 3 mice per group). **B**, adductor muscles in the upper limb were harvested at day 3 and analyzed for F4/80+ myeloid accumulation (brown and arrows) at the perivascular space of collateral arteries. Eosin staining was performed to show the structures. (n = 3 mice per group and *p<0.05). **C**, ischemic tibialis anterior muscles were harvested at day 3 and analyzed for mRNA expression of intercellular adhesion molecule 1 (*icam1*), vascular cell adhesion molecule 1 (*vcam1*) and monocyte chemotactic protein-1 (MCP-1 (*ccl2*)) by real-time polymerase chain reaction. Ribosomal 18S and *hprt* were used as internal controls. Relative expression for WT is shown (n = 3 mice per group). **D**, vascular endothelial growth factor (VEGF) expression was analyzed by Western blotting of protein lysate from ischemic tibialis anterior muscle at day 7. Alpha tubulin is shown as control. Densitometry analysis is shown (n = 3 mice per group). All data shown are mean+SE (*p<0.05).

Since inflammatory response also plays an important role in angiogenesis and arterial remodeling 1,6,42–44, we next examined the infiltration of inflammatory cells in ischemic tissues of WT and Cat-Tg mice. Immunohistochemistry showed that F4/80 positive macrophage accumulation in the ischemic region of gastrocnemius muscles was decreased in Cat-Tg mice compared with WT mice at day 7 after injury (Figure 4A). Furthermore, perivascular accumulation of F4/80 positive myeloid cells in the upper limbs after ischemia was also decreased in Cat-Tg mice (Figure 4B).

Since accumulation of macrophages has been shown to be mediated by adhesion molecule and chemokine expression in injured tissues [1], [6], [43], [45], we next examined the expression of vascular cell adhesion molecule 1 (VCAM-1), intercellular adhesion molecule 1 (ICAM-1), and monocyte chemotactic protein-1 (MCP-1) in WT and Cat-Tg mice. Figure 4C shows that mRNAs for VCAM-1 and MCP-1, but not ICAM-1, were significantly reduced in ischemic tissues of Cat-Tg mice compared to WT mice. This was associated with a decrease in Ser536 phosphorylation of p65 nuclear factor-κB (NFκB), which is required for its activity, in Cat-Tg mice (Figure S2). Moreover, protein expression of VEGF, which is mainly secreted by the infiltrated macrophage [46], in ischemic tissues was markedly decreased by endothelial catalase overexpression (Figure 4D). Thus, these results suggest that H_2_O_2_ in ECs increases VCAM-1 and MCP-1 expression, thereby promoting macrophage accumulation in the ischemic tissues and perivascular regions, which in turn increases angiogenesis and arteriogenesis.

### Ischemia-induced increase in circulating vascular progenitor cells is inhibited in Cat-Tg mice

**Figure 5:**
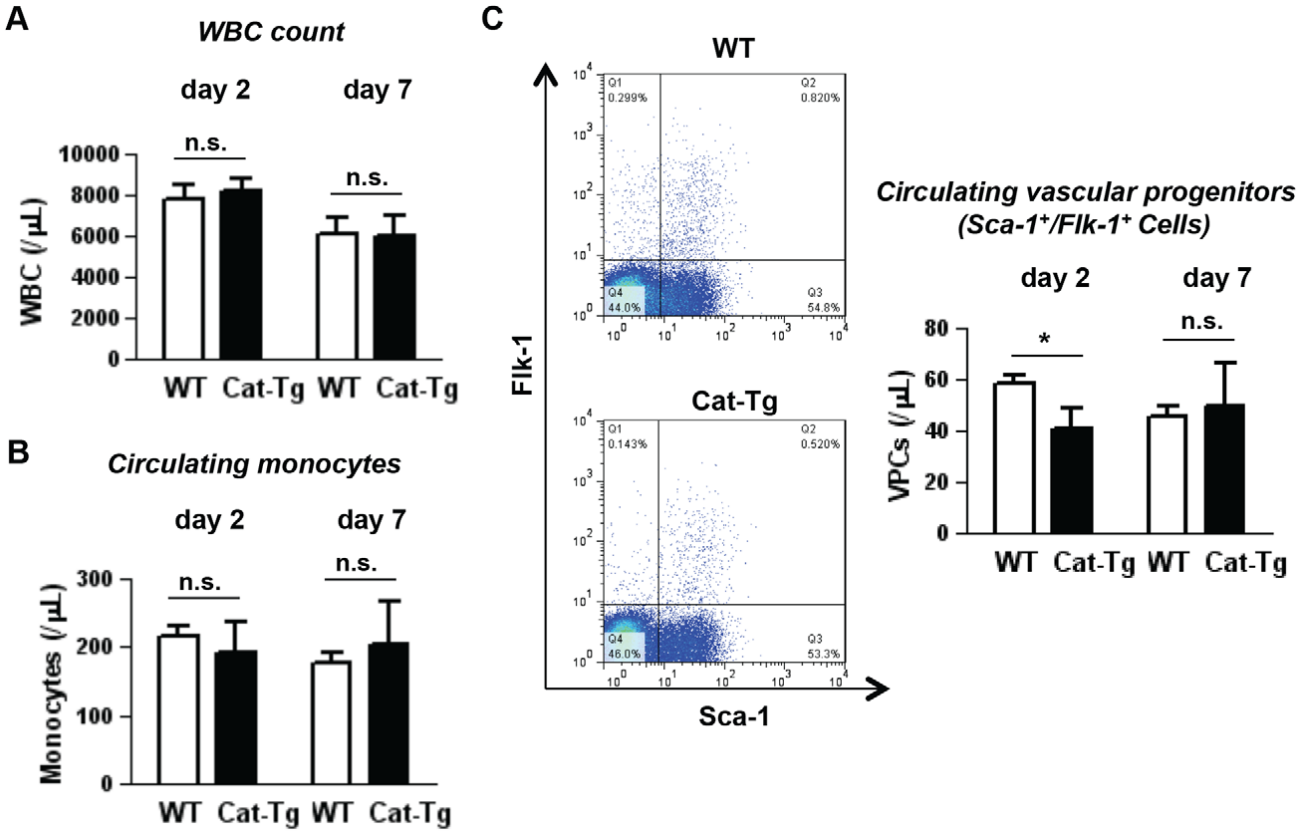
Tie2-driven catalase overexpression affects circulating progenitor cell level. The levels of white blood cells, monocytes and vascular progenitors (Sca1^+^/Flk1^+^ cells) were analyzed in the peripheral blood at indicated time points. Representative plots of vascular progenitors at day 2 are shown. All data shown are mean+SE (n = 4 mice per group and *p<0.05).

We next examined the levels of circulating leukocytes and monocytes as well as vascular progenitor cells (Sca-1^+^/Flk^+^) after hindlimb ischemia in WT and Cat-Tg mice. FACS analysis reveals that there was no significant difference in hindlimb ischemia-induced increase in the numbers of white blood cells (Figure 5A) and monocytes (Figure 5B) in peripheral blood between WT and Cat-Tg mice. However, circulating Sca1^+^/Flk1^+^ vascular progenitor cell numbers were significantly reduced on day 2 after hindlimb ischemia in Cat-Tg mice, while this difference was not observed in the later phase on day 7 (Figure 5C). Of note, catalase is not overexpressed in BM myeloid cells in Cat-Tg mice [27]. Taken together, these results suggest that H_2_O_2_ derived from Tie2+ vascular ECs in BM at least in part may promote mobilization of vascular progenitor cells, but not inflammatory cells, after ischemic injury.

### Intracellular H_2_O_2_ production and eNOS phosphorylation in ischemic tissues are inhibited in Cat-Tg mice

**Figure 6:**
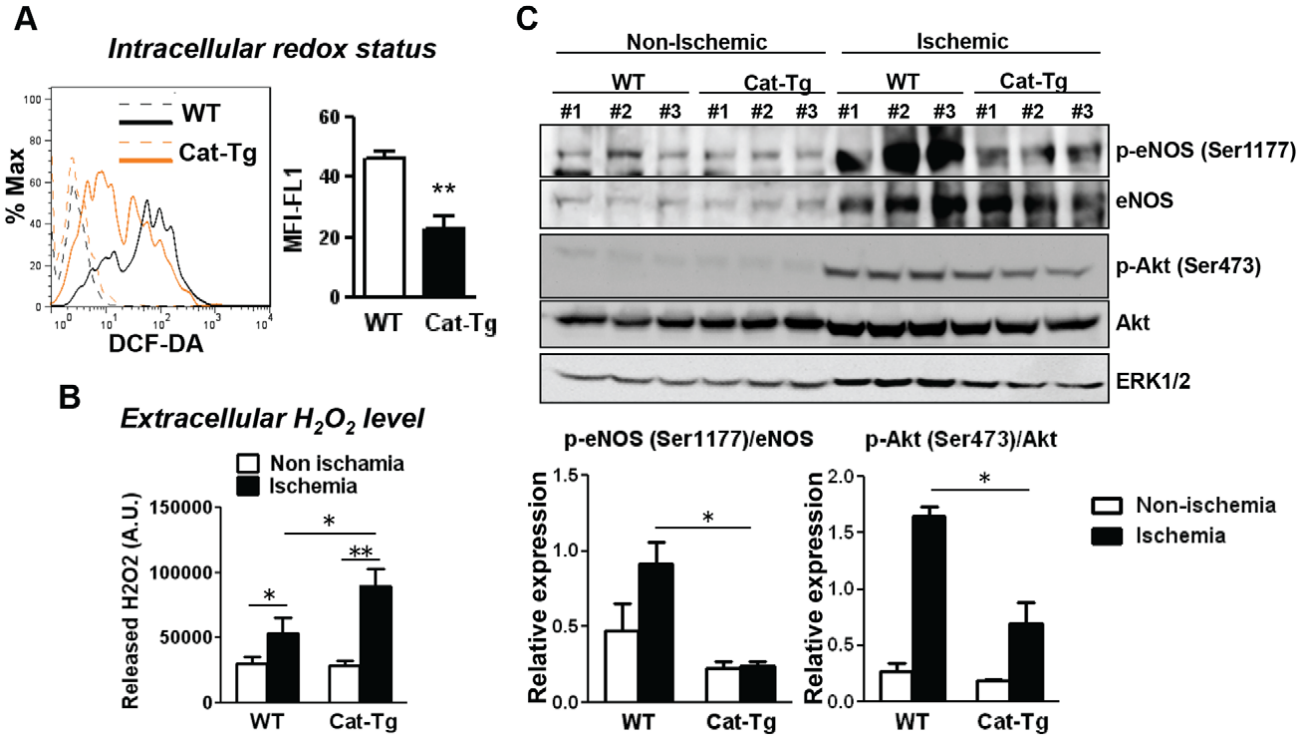
Intracellular H_2_O_2_ in endothelial cells regulate endothelial nitric oxide synthase activation *in vivo*. **A**, intracellular redox status was measured by 2′,7′-dichlorfluorescein-diacetate (DCF-DA) staining in gated CD31^+^/CD45^−^ population of collagenase-digested ischemic muscles at day 3. The dotted lines indicate the background signals without DCF-DA. **B**, ischemic muscles from Wild-type (WT) and Tie2-driven catalase transgenic (Cat-Tg) mice at day 3 were isolated and incubated. Their H_2_O_2_ production was measured by Amplex Ultra Red assay. **C**, harvested ischemic and non-ischemic muscles at day 3 were analyzed for protein expression of phosphorylated and total form of endothelial nitric oxide synthase (eNOS), Akt and ERK1/2 (as control) by Western analysis. Densitometry analysis in activation (phosphorylation) of each protein is shown. All data shown are mean+SE (n = 3 mice per group, *p<0.05, **p<0.01 and ***p<0.001).

Endothelial nitric oxide synthase (eNOS) is a key regulator of angiogenesis [47], and H_2_O_2_ is shown to increase eNOS expression and activity through its phosphorylation at Ser1177 [48], [49], thereby promoting NO production. We first examined the intracellular redox status in ECs and extracellular H_2_O_2_ levels in ischemic tissues during angiogenesis. To estimate intracellular H_2_O_2_ levels, we employed DCF-DA staining on collagenase-digested ischemic tissues combined with cell surface marker staining, which have been recently used to demonstrate redox status of angiogenic ECs in mice [34]. We found that intracellular oxidation state in CD31^+^/CD45^−^ ECs from ischemic muscles was significantly reduced in Cat-Tg mice (Figure 6A). In contrast, extracellular H_2_O_2_ production from the ischemic tissue, as measured by Amplex Red assay, was even higher in Cat-Tg mice (Figure 6B). Under this condition, Cat-Tg mice exhibited a significant decrease in ischemia-induced eNOS phosphorylation at Ser1177 without affecting eNOS expression in ischemic tissues. Ischemia-induced phosphorylation of Akt at Ser473, an upstream kinase for p-eNOS (Ser1177) [50], but not total Akt protein, was also significantly inhibited in ischemic tissues from Cat-Tg mice (Figure 6C). Thus, intracellular H_2_O_2_ derived from ECs activates at least Akt-eNOS-NO pathway to promote ischemia-induced angiogenesis.

### Endothelium-dependent relaxation of resistant vessels is impaired in Cat-Tg mice

**Figure 7:**
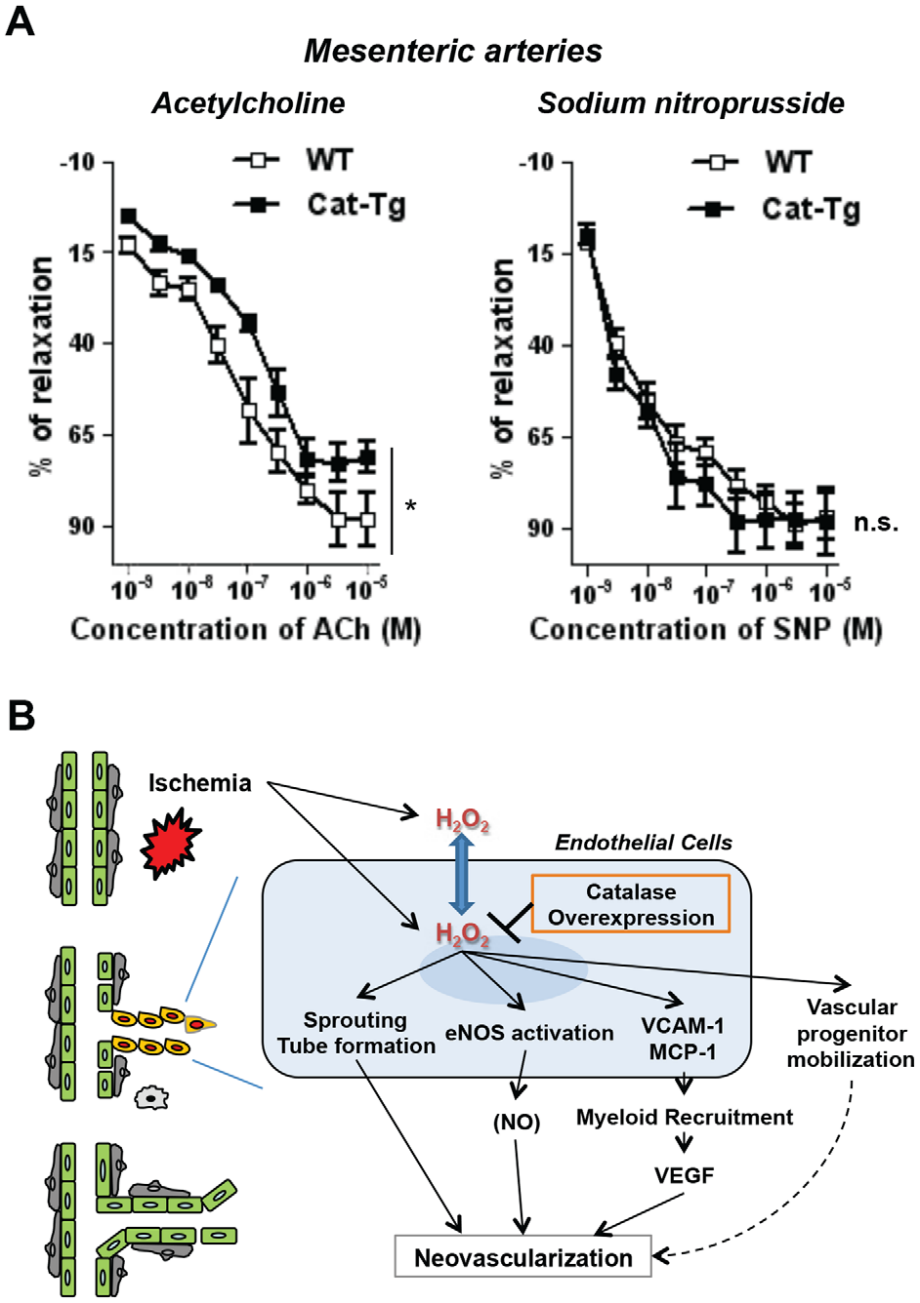
Endothelial catalase overexpression blunts endothelium-dependent relaxation of resistant vessels. **A**, the first branches mesenteric arteries were harvested from Wild-type (WT) and Tie2-driven catalase transgenic (Cat-Tg) mice and assessed for endothelium-dependent or – independent relaxation by acetylcholine or sodium nitroprusside, respectively (n = 4 per group and *p<0.05). Data shown are mean+SE. **B**, a proposed model for the role of endogenous H_2_O_2_ in endothelial cells during ischemia-induced neovascularization. Tissue ischemia induces endogenous reactive oxygen species production including hydrogen peroxide (H_2_O_2_) intracellularly and extracellularly for endothelial cells. Intracellular H_2_O_2_, which can be reduced by Tie2-driven catalase overexpression in this study, promote crucial neovascular signaling regulating endothelial sprouting and tube formation, endothelial nitric oxide synthase (eNOS) activation as well as the expression of vascular adhesion molecule (VCAM)-1 and monocyte chemoattractant protein (MCP)-1. Endothelial H_2_O_2_ could be involved in vascular progenitor mobilization. H_2_O_2_ is thought to be diffusible across cellular membrane (blue arrow). Myeloid recruitment, vascular endothelial growth factor (VEGF) and potential nitric oxide (NO) production are regulated by endogenous H_2_O_2_ in endothelial cells during neovascularization.

To determine further the vascular H_2_O_2_-dependent endothelial function, we examined the endothelium-dependent relaxation of mesenteric resistant arteries from WT and Cat-Tg mice. Figure 7 shows that endothelial overexpression of catalase significantly blunted acetylcholine (Ach)-induced endothelium-dependent vasorelaxation without affecting sodium nitroprusside (SNP)-induced endothelium-independent vessel relaxation. Of note, Ach-induced endothelium-dependent relaxation of mesenteric arteries was inhibited by L-NAME, an inhibitor of NOS [51], by 69% in our experimental condition (p<0.05, data not shown). These results suggest that endogenous ECs-derived H_2_O_2_ is in part involved in endothelium-dependent relaxation of resistant vessels in response to Ach.

## Discussion

Using endothelium-specific catalase overexpressing transgenic mice [27], [28], the present study provides the direct evidence that endogenous H_2_O_2_ in ECs plays a critical role in reparative neovascularization by promoting angiogenesis, collateral remodelling, and myeloid cell recruitment to ischemic tissues. Mechanistically, Cat-Tg mice show a decrease in eNOS activation as well as VCAM-1 and MCP-1 expression in ischemic tissues. Endothelial H_2_O_2_ is also involved in the early phase of vascular progenitor cell mobilization from BM in response to hindlimb ischemia. Moreover, experiments with isolated vessels reveal that H_2_O_2_ in ECs contributes to vessel sprouting and tube elongation as well as endothelium-dependent relaxation of resistant vessels (Figure 7B).

We previously characterized the Cat-Tg mice driven by Tie2 promoter and demonstrated that human catalase protein is overexpressed in the endothelium, but not non-vascular cells [27]. Since Tie2 might be expressed in myeloid cells [52], we measured catalase protein expression in BM cells, but found no difference between WT and Cat-Tg mice [27]. Here we demonstrate that ischemia-induced neovascularization and intracellular oxidation state in ECs isolated from ischemic tissues, as measured by DCF-DA with FACS analysis, are significantly reduced by overexpression of catalase in ECs. The method to measure intracellular redox status in ECs isolated from the tissue has been recently reported [34]. Moreover, VEGF-induced increase in DCF fluorescence was inhibited in cultured ECs derived from Cat-Tg mice. By contrast, extracellular H_2_O_2_ production from ischemic muscles, as measured by Amplex Red assay, was rather enhanced in Cat-Tg mice after hindlimb ischemia. This may be due to the possibility that other cellular sources such as inflammatory cells, vascular smooth muscle cells, or skeletal muscles in ischemic tissues may produce high levels of H_2_O_2_ in response to ischemia, which might mask the localized and small fraction of intracellular H_2_O_2_ produced from ECs. Consistent with our data, mice with overexpression of catalase in myeloid cells, which exhibit impaired post-ischemic neovascularization, do not show decrease in total H_2_O_2_ production in ischemic muscles, while isolated macrophages from these mice show less H_2_O_2_ production than those from control mice, assessed by Amplex Red assay [26]. Given that H_2_O_2_ is stable and highly diffusible molecule, H_2_O_2_ derived from myeloid cells recruited to the ischemic tissues may enter the ECs membranes in part through the aquaporins [53], [54] and function as an environmental cue to regulate EC function. Developing new probes to detect and quantify H_2_O_2_ with high degree of spatial and temporal resolution in intact tissue *in vivo* is essential to address this possibility. Although the sources of H_2_O_2_ are likely multiple, these results suggest that increase in intracellular H_2_O_2_ in ECs is required for reparative neovascularization in response to tissue ischemia.

In this study, CD31^+^ capillary staining in the early phase of post-ischemic neovascularization reveals that H_2_O_2_ in ECs regulate morphology of newly formed vessels in the ischemic tissue. The overexpression of catalase results in disorganized microvasculature formation at day 3 and causes substantial decrease in the number of microvessels at day 7. Aortic ring assays in Matrigel, an *ex vivo* model of angiogenesis, also demonstrate impaired vascular formation, as vessel sprouting and tube elongation are inhibited by endothelial-specific catalase overexpression. Consistent with our results, Craige et al. [24] recently reported that transgenic mice with endothelial specific overexpression of Nox4, which mainly generates H_2_O_2_ rather than O_2_^•−^, promote angiogenesis in response to hindlimb ischemia. In addition, global and tamoxifen-inducible Nox4^−/−^ mice showed reduced post-ischemic angiogenesis [25], however, this study did not provide the specific cell types responsible for endogenous Nox4-mediated responses *in vivo*. Thus, the present study provides the direct evidence that endogenous H_2_O_2_ in ECs is required for new vessel formation in response to ischemia.

Inflammatory cell recruitment is critical for post-ischemic angiogenesis and collateral vessel remodeling [1], [6], [42], [46]. Here we demonstrate that F4/80-positive myeloid cell recruitment into the site of neovascularization is impaired in Cat-Tg mice, which is associated with a decrease in VEGF, VCAM-1 and MCP-1 expression in ischemic muscles. It has been reported that macrophage-derived VEGF contributes to angiogenesis and arteriogenesis after tissue ischemia [46] and that inflammatory signals increase VCAM-1 and MCP-1 expression through ROS-dependent NFκB activation in ECs [55]. Consistent with this, Ser536 phosphorylated NFκB p65 in the ischemic tissue is markedly reduced in Cat-Tg mice. VCAM-1 regulates leukocytes adhesion and trans-endothelial migration [56], and MCP-1 is a major chemokine that attracts monocytes, and thus promoting post-ischemic neovascularization [42]. Moreover, endothelial MCP-1 expression facilitates the maturation of newly formed microvessels [57], which may explain why Cat-Tg mice exhibit an abnormal morphology in early neovessels. Given that catalase is not overexpressed in whole BM cells in Cat-Tg mice, reduction of myeloid cells recruitment is likely due to the decrease in H_2_O_2_ production in ECs, but not BM cells. This notion is further supported by our observation that circulating levels of total leukocytes or monocytes in response to ischemia are not different between WT and Cat-Tg mice. These results suggest that endothelium-derived H_2_O_2_ activates NFκB, thereby increasing redox-sensitive inflammatory genes such as VCAM-1 and MCP-1 in ischemic tissues, which may facilitate recruitment of inflammatory cells after ischemic injury. However, we cannot exclude the possibility that the reduction of inflammatory cell levels or population in ischemic tissues may also contribute the decrease in NFkB activation. Of note, Hodara et al. [26] reported that mice with catalase overexpression in myeloid cells also impair macrophage infiltration into ischemic tissues after hindlimb ischemia. Taken together, these findings indicate that H_2_O_2_ derived from both ECs and inflammatory cells may play an important role for post-ischemic inflammatory recruitment and neovascularization. This is consistent with previous reports that Nox2-derived ROS in BM cells [11] and Nox4-derived H_2_O_2_ in ECs [24] promote neovascularization after hindlimb ischemia.

Not only inflammatory cells but also vascular progenitor cells are mobilized from BM to circulation after hindlimb ischemia, which contributes to reparative angiogenesis [2]–[5]. In this study, we found that ischemia-induced Sca-1^+^/Flk1^+^ progenitor cells in peripheral blood is significantly reduced at day 2, but not at day 7, after injury in Cat-Tg mice. This result suggests that endothelial H_2_O_2_ may promote vascular progenitor cell mobilization in the early phase after hindlimb ischemia. Consistent with this, we previously reported that NADPH oxidase-derived ROS are required for BM progenitor cells mobilization at day 3 after ischemia [11]. By contrast, transgenic mice overexpressing catalase in myeloid cells have no effects on the number of endothelial progenitor cells in peripheral blood or BM at 3 days after ischemia [26]. In addition, chronic exercise for three weeks rather increases circulating endothelial progenitor cells in Cat-Tg mice, but not in control mice [58]. Thus, these findings indicate that elevation of H_2_O_2_ in ECs, but not myeloid cells, in the BM microenvironment in response to tissue ischemia may stimulate acute reparative mobilization of vascular progenitor cells. However, when H_2_O_2_ is produced at excessive level or long term after ischemic injury or chronic exercise, it may have a negative impact on mobilizing BM progenitor cells. This is consistent with the notion for doubled-edge role of ROS in which physiological levels can serve as signaling molecules to promote vascular integrity, whereas excess ROS levels in pathological conditions contribute to stem/progenitor dysfunction and impaired neovascularization [59]. The molecular mechanism of how H_2_O_2_ derived from ECs regulates progenitor cell function in the BM after ischemic injury requires further investigation.

Previous studies have shown that eNOS plays an important role in post-ischemic angiogenesis [47] and that H_2_O_2_ increases activity and expression of eNOS in cultured ECs [49], [60]. Our current study demonstrates that hindlimb ischemia-induced eNOS phosphorylation at Ser1177 is inhibited in Cat-Tg mice. In line with our data, previous studies show that eNOS protein is upregulated in cultured ECs derived from endothelial Nox4 overexpressing mice [24], and that it is downregulated in isolated carotid artery derived from Nox4 deficient mice [25]. Moreover, exercise-induced upregulation of eNOS protein in aorta and heart is inhibited in Cat-Tg mice [28]. Thus, the present study provides the first evidence that endothelial H_2_O_2_ is involved in eNOS phosphorylation at Ser1177 in ischemic muscles after hindlimb ischemia *in vivo*. Of note, above findings demonstrating the H_2_O_2_-eNOS-NO axis is in contrast to the general notion for the decreasing NO bioavailability by the pathological increase in O_2_^•−^. This discrepancy is likely due to the fact that O_2_^•−^, but not H_2_O_2_, highly reacts with NO to produce peroxynitrite, thereby inducing endothelial dysfunction. In the present study, catalase overexpression significantly inhibited Akt phosphorylation to a lesser extent than p-eNOS. This result suggests that ischemia-induced endothelial H_2_O_2_ activates eNOS by phosphorylating at Ser1177 through Akt-dependent and -independent manner, thereby promoting NO production and post-ischemic angiogenesis *in vivo*. Thus, it is possible that H_2_O_2_-induced eNOS phosphorylation at Ser1177 in ischemic tissues may be mediated through activation of other redox-sensitive kinases such as AMP kinase [61] and Src kinase [62], or through redox-mediated inactivation of protein phosphatases such as PTEN [63] or PP2A [64] which is shown to dephosphorylate eNOS. Additional targets of endothelial H_2_O_2_ involved in postnatal angiogenesis should be clarified in future study.

To demonstrate further the role of endothelial H_2_O_2_ in endothelial function, we show that endothelium-dependent relaxation of mesenteric arteries in response to Ach, but not endothelium-independent vasorelaxation to a NO donor, is impaired in Cat-Tg mice. By contrast, previous study demonstrates that Ach-induced endothelium-dependent relaxation of aorta is not affected in Cat-Tg mice [29]. These results suggest that Ach-induced endothelium-dependent vasodilation is in part dependent on H_2_O_2_ in the small resistant vessels (mesenteric artery), but not in the large conductance vessels (aorta). It has been reported that H_2_O_2_ functions as an endothelium-derived hyperpolarizing factor (EDHF) 65–67 in mouse or human mesenteric arteries to induce vasorelaxation [68], and that source of this H_2_O_2_ is proposed to be NOS [66], mitochondrial ROS [69], and NADPH oxidase [70]. Thus, current findings indicate that ECs-derived H_2_O_2_ stimulates endothelium-dependent vasodilation in resistant vessels, thereby regulating tissue perfusion and improving tissue ischemia.

In this study, we demonstrate a positive role of ROS, particular H_2_O_2_, in post-ischemic reparative neovascularization, which is consistent with previous reports that Nox2-derived ROS [11], [30] or Nox4-derived H_2_O_2_ [24], [25] or H_2_O_2_ derived from myeloid cells [26] are required for this response. However, excess amount of H_2_O_2_ in pathological conditions has a negative impact on endothelial function, neovascularization and tissue repair. For examples, Nox2 deficiency rescues impaired post-ischemic angiogenesis in type1 diabetic mice [12], old mice exposed to tobacco smoke [14] or atherosclerotic mice [13], while it reduces neovascularization in young, healthy mice [11], [30]. Moreover, knockout mice for antioxidant enzymes such as extracellular SOD (SOD3) [71] or Cu/Zn SOD (SOD2) [72] or GPx-1 [73] show impaired neovascularization due to excess ROS levels leading to apoptotic ECs or EPCs. These findings are consistent with the “redox window” or “oxidative window” concept suggesting that optimal levels of H_2_O_2_ are required for signaling and normal biological function, while excess or insufficient levels of ROS are associated with cellular dysfunction [74]–[76]. In addition, we found that VEGF level is markedly reduced in the ischemic tissue in Cat-Tg mice. It is possible that reduced VEGF can explain the impaired angiogenesis and decreased VEGF-dependent signaling in ischemic tissues in these mice. However, results obtained with aortic ring assay conducted under VEGF-stimulated condition as well as vascular relaxation studies suggest that endothelial H_2_O_2_ can regulate endothelial functions, independent of VEGF. Moreover, although findings obtained using Cat-Tg mice may provide a consequence of loss of H_2_O_2_-dependent signaling, we cannot eliminate the possibility that they may also reflect the indirect changes in intracellular redox state induced by decrease in intracellular H_2_O_2_ in ECs.

In summary, our findings demonstrate that H_2_O_2_ at appropriate level in ECs activates specific signaling pathways leading to angiogenesis, inflammatory cell recruitment, and vascular progenitor cell mobilization, which contribute to reparative neovascularization in response to ischemic injury. Moreover, endothelial H_2_O_2_ is involved in endothelium-dependent relaxation in resistant vessels to preserve endothelial function. Redox-regulation in ECs is a potential therapeutic strategy for angiogenesis-dependent ischemic cardiovascular diseases.

## Supporting Information

Figure S1 **Endothelial-specific catalase overexpression in mice.** **A**, aortas was harvested from wild-type (WT) and Tie2-driven catalase transgenic (Cat-Tg) mice and human catalase (hCatalase) mRNA was confirmed by real-time polymerase chain reaction. **B**, endothelial catalase overexpression was confirmed in primary cultured endothelial cells from mice. **C**, mouse endothelial cells were isolated from WT and Cat-Tg mice and cultured in the growth media. Intracellular redox status was assessed by dichlorofluorescein diacetate (DCF-DA) staining under VEGF stimulation. Images (63x objective) were taken by laser confocal microscopy.
(TIF)

Figure S2 **Endothelial catalase overexpression reduces NFκB activation in the ischemic tissue.** Harvested ischemic muscles at day 3 were measured for phosphorylation of NFκB p65 at Ser536, which is shown to correlate with NFκB activation and its total protein by Western analysis.
(TIF)
