# Supplementary figures and images for: Critical Role of Endothelial Hydrogen Peroxide in Post-Ischemic Neovascularization
Source: PLoS One. 2013 Mar 5;8(3):e57618. doi: 10.1371/journal.pone.0057618 (PMC3589391; doi:10.1371/journal.pone.0057618)

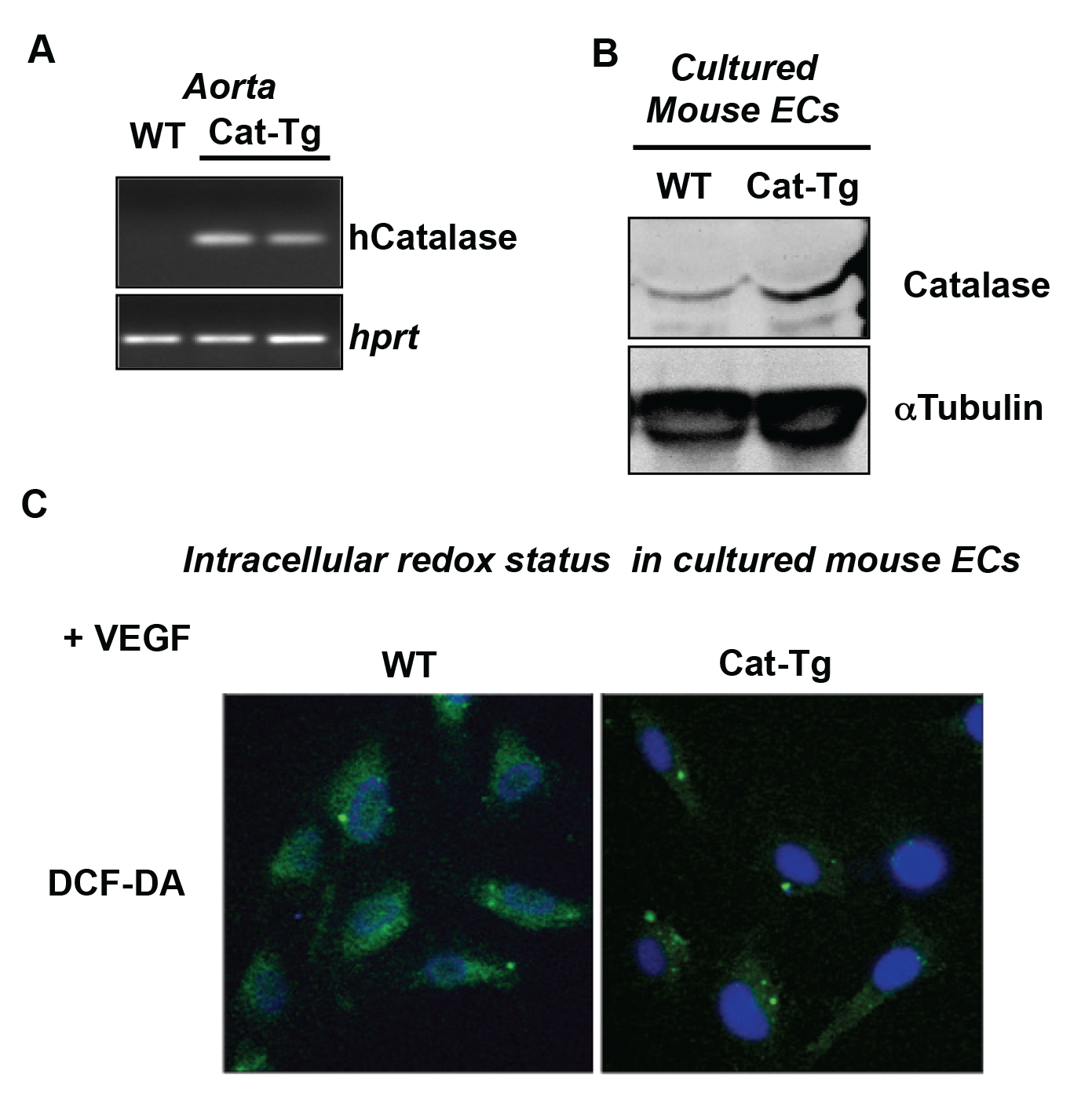

Supplement: Figure S1 — Endothelial-specific catalase overexpression in mice. A, aortas was harvested from wild-type (WT) and Tie2-driven catalase transgenic (Cat-Tg) mice and human catalase (hCatalase) mRNA was confirmed by real-time polymerase chain reaction. B, endothelial catalase overexpression was confirmed in primary cultured endothelial cells from mice. C, mouse endothelial cells were isolated from WT and Cat-Tg mice and cultured in the growth media. Intracellular redox status was assessed by dichlorofluorescein diacetate (DCF-DA) staining under VEGF stimulation. Images (63x objective) were taken by laser confocal microscopy. (TIF) [file pone.0057618.s001.tif]

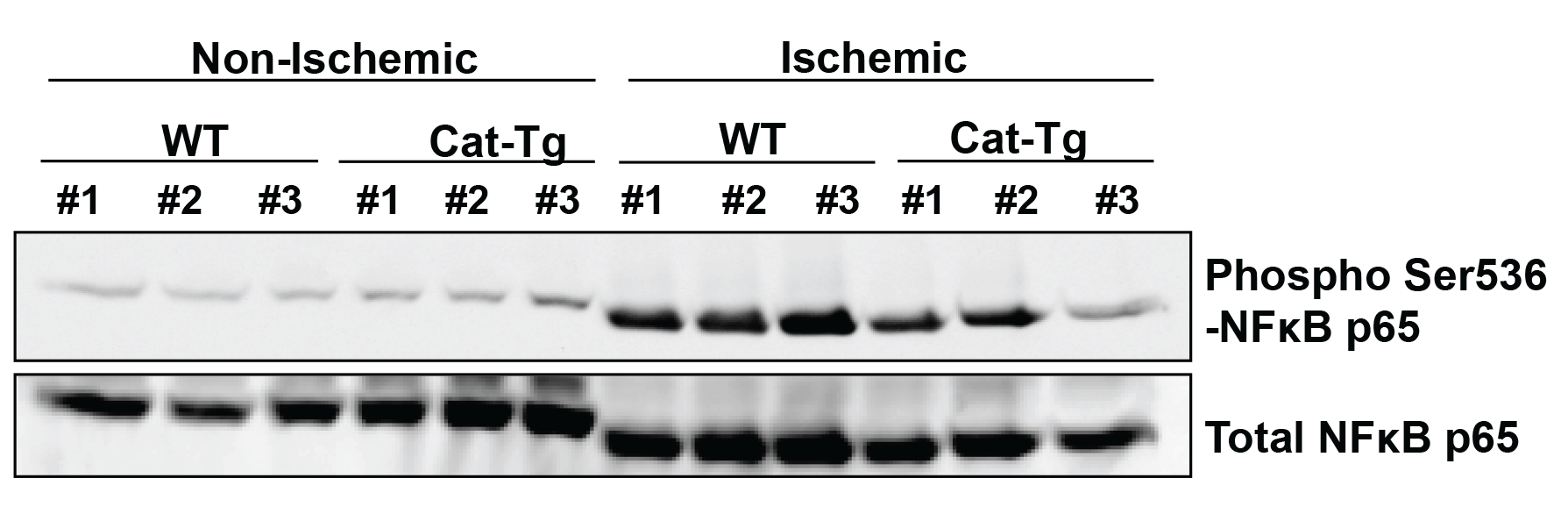

Supplement: Figure S2 — Endothelial catalase overexpression reduces NFκB activation in the ischemic tissue. Harvested ischemic muscles at day 3 were measured for phosphorylation of NFκB p65 at Ser536, which is shown to correlate with NFκB activation and its total protein by Western analysis. (TIF) [file pone.0057618.s002.tif]
